# Dihydrolipoic acid protects against lipopolysaccharide-induced behavioral deficits and neuroinflammation via regulation of Nrf2/HO-1/NLRP3 signaling in rat

**DOI:** 10.1186/s12974-020-01836-y

**Published:** 2020-05-25

**Authors:** Hetao Bian, Gaohua Wang, Junjie Huang, Liang Liang, Yage Zheng, Yanyan Wei, Hui Wang, Ling Xiao, Huiling Wang

**Affiliations:** Department of Psychiatry, Renmin Hospital of Wuhan University, Jiefang Road 238#, Wuhan, 430060 Hubei PR China

**Keywords:** Dihydrolipoic acid, Neuroinflammation, NLRP3, Lipopolysaccharide, Depression

## Abstract

**Background:**

Recently, depression has been identified as a prevalent and severe mental disorder. However, the mechanisms underlying the depression risk remain elusive. The neuroinflammation and NLRP3 inflammasome activation are known to be involved in the pathology of depression. Dihydrolipoic acid (DHLA) has been reported as a strong antioxidant and exhibits anti-inflammatory properties in various diseases, albeit the direct relevance between DHLA and depression is yet unknown. The present study aimed to investigate the preventive effect and potential mechanism of DHLA in the lipopolysaccharide (LPS)-induced sickness behavior in rats.

**Methods:**

Adult male Sprague–Dawley rats were utilized. LPS and DHLA were injected intraperitoneally every 2 days and daily, respectively. Fluoxetine (Flu) was injected intraperitoneally daily. PD98059, an inhibitor of ERK, was injected intraperitoneally 1 h before DHLA injection daily. Small interfering ribonucleic acid (siRNA) for nuclear factor erythroid 2-like (Nrf2) was injected into the bilateral hippocampus 14 days before the DHLA injection. Depression-like behavior tests were performed. Western blot and immunofluorescence staining detected the ERK/Nrf2/HO-1/ROS/NLRP3 pathway-related proteins.

**Results:**

The DHLA and fluoxetine treatment exerted preventive effects in LPS-induced sickness behavior rats. The DHLA treatment increased the expression of ERK, Nrf2, and HO-1 but decreased the ROS generation levels and reduced the expression of NLRP3, caspase-1, and IL-1β in LPS-induced sickness behavior rats. PD98059 abolished the effects of DHLA on preventive effect as well as the levels of Nrf2 and HO-1 proteins. Similarly, Nrf2 siRNA reversed the preventive effect of DHLA administration via the decreased expression of HO-1.

**Conclusions:**

These findings suggested that DHLA exerted a preventive effect via ERK/Nrf2/HO-1/ROS/NLRP3 pathway in LPS-induced sickness behavior rats. Thus, DHLA may serve as a potential therapeutic strategy for depression.

## Background

Depression is the most common psychiatric mood disorder globally. Currently, the limited therapies available for the treatment of this condition mainly target the monoamine levels. However, this approach is not efficient for many patients [1]. The mechanisms underlying depression are complicated and largely unknown. Therefore, the knowledge of this mechanism of depression would aid the development of effective treatment.

Accumulating evidence revealed a close link between inflammation and major depression disorder [2, 3]. The nod-like receptor pyrin-containing pyrin domain 3 (NLRP3) inflammasome is one of the most widely studied inflammasomes [4]. After NLRP3 inflammasome is assembled and activated, it activates caspase-1 by proteolytic cleavage, which in turn, converts pro-IL-1β into bioactive IL-1β, leading to inflammatory responses in bodies [5, 6]. Reactive oxygen species (ROS) is a normal metabolic product of redox reactions. The excessive level of ROS would damage the integrity of cells and result in dysfunction of the tissues via peroxidation of lipids, proteins, mitochondria, and DNA of cells [7, 8]. Despite that the specific regulatory mechanism of NLRP3 inflammasome activation is unclear, ROS has been frequently reported to be correlated with NLRP3 inflammasome activation [9, 10]. Also, it regulates the expression and/or activation of NLRP3 inflammasome in several diseases, including intestinal inflammation and cardiovascular disease.

Microglia, as resident immune cells which fulfill different tasks, are mainly involved in the inflammatory response and in maintaining homeostasis within the central nervous system (CNS). Microglial activation is the principal component of neuroinflammation in the CNS, the function of microglial cells in the pathophysiology of depression has attracted more and more attention [11].

Nuclear factor erythroid 2-like (Nrf2) is a primary element of transcription and has emerged as a potential therapeutic target for inflammatory disorders [12]. Heme oxygenase-1 (HO-1) is an enzyme that exerts anti-inflammatory and antioxidant stress effects [13]. Nrf2 is a critical modulator of the expression of HO-1 [14, 15]. Recent studies have focused on the Nrf2/HO-1 approach with respect to anti-inflammation [16, 17]. These findings indicated that the Nrf2/HO-1 signaling pathway plays a critical role in anti-inflammatory activities. Other studies have shown that the activated Nrf2/HO-1 signaling pathway may counteract the intracellular production of ROS [18].

Dihydrolipoic acid (DHLA) is a reduced form of α-lipoic acid (LA) that can decrease oxidative stress and act as a strong antioxidant. DHLA also possesses anti-inflammatory properties [19]. Hitherto, the preventive effect of DHLA has not been explored. Therefore, in the present study, we sought to investigate the preventive effect of DHLA in the LPS-induced sickness behavior animal model and whether ERK/Nrf2/HO-1/ROS/NLRP3 pathway is involved in the preventive effect of DHLA.

## Methods

### Animals

Adult male Sprague–Dawley (SD) rats (weight, 200–220 g) were purchased from Hunan SJA Laboratory Animal Co., Ltd (Hunan, China) and housed in a 12 h dark and light cycle at room temperature (18–22 °C) with free access to water and food. All procedures involving animals were approved and carried out according to the guidelines of the Institutional Animals Care Committee of Renmin Hospital of Wuhan University.

### Experimental design

**Fig. 1:**
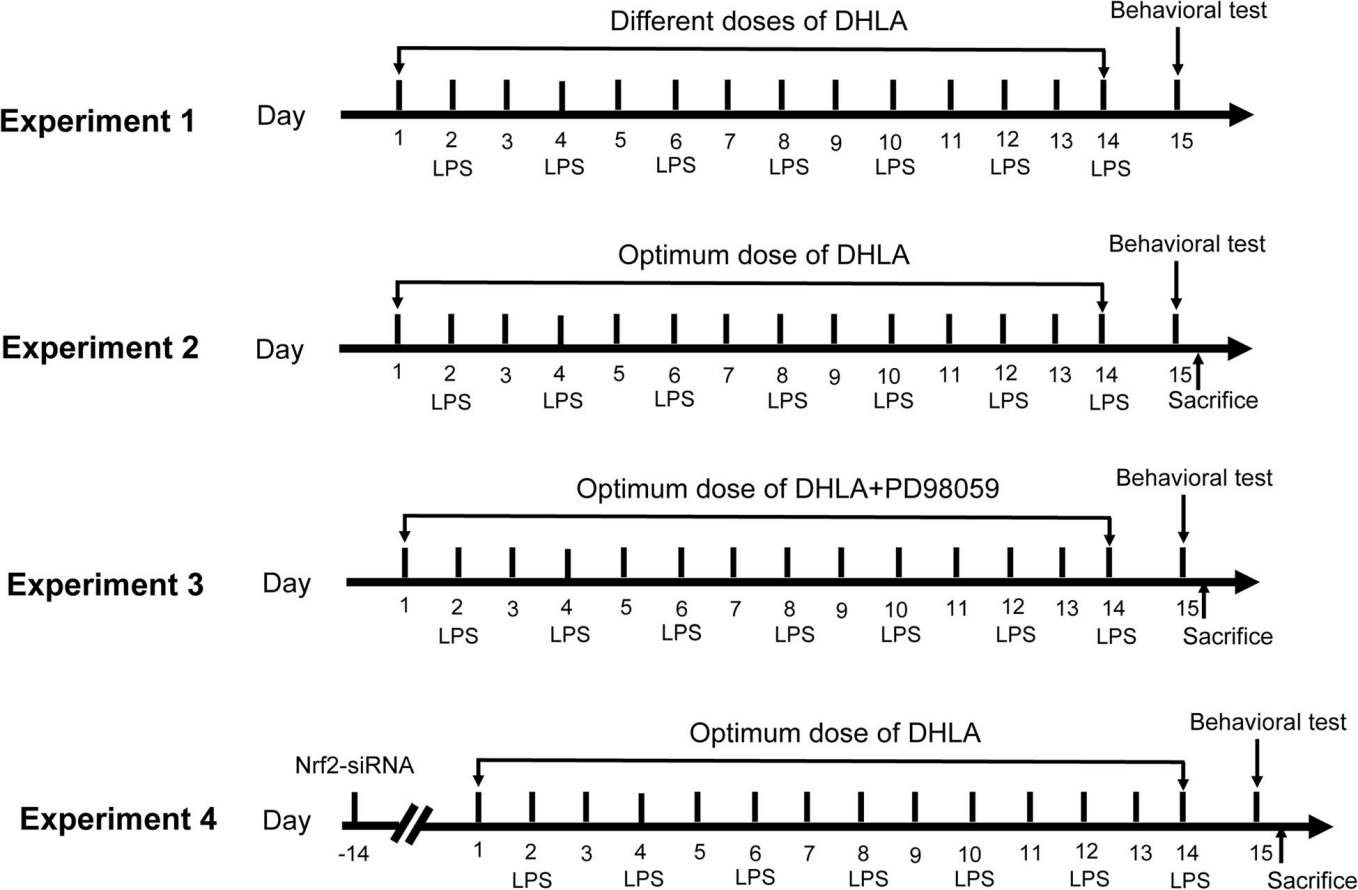
Experimental procedures and timeline. Experiment 1: Effects of different doses of DHLA on treatment of sickness behavior rats. Experiment 2: Mechanism study of DHLA on sickness behavior rats. Experiment 3: ERK inhibitors are used to test the mechanism of DHLA on sickness behavior rats. Experiment 4: Nrf2 inhibitors are used to test the mechanism of DHLA on sickness behavior rats

This study consisted of four experiments as shown in Fig. 1.

Experiment 1: Rats were randomly divided into six groups (*n* = 6/group): Control, Lipopolysaccharide (LPS) + vehicle, LPS + DHLA (15 mg/kg, 30 mg/kg, 60 mg/kg), LPS+ fluoxetine (Flu) group. The evaluation of the body weight, open field test (OFT), and forced swim test (FST) was used to assess the anti-depression effects of DHLA. Based on the body and behavioral tests, 30 mg/kg DHLA-treated group was selected for the subsequent experiments.

Experiment 2: Rats were randomly divided into four groups (*n* = 9/group): Control, LPS, LPS+ vehicle, and LPS + DHLA (30 mg/kg). The expression of ERK, Nrf2, and HO-1 was detected by Western blot, while that of ROS was tested by flow cytometry (*n* = 6/group). Immunofluorescence staining assessed the expression of HO-1 (*n* = 3/group). The test of body weight, OFT, and FST was used to assess the anti-depression effects of DHLA.

Experiment 3: Rats were randomly divided into six groups (*n* = 6/group): Control, LPS, LPS+ vehicle, LPS + DHLA (30 mg/kg), LPS + DHLA (30 mg/kg) + DMSO, LPS + DHLA (30 mg/kg) + PD98059. The expression of ERK, Nrf2, HO-1, NLRP3, caspase-1, and IL-1β was detected by Western blot. The test of body weight, OFT, and FST was used to assess the anti-depression effects of DHLA.

Experiment 4: Rats were randomly divided into six groups (*n* = 6/group): Control, LPS, LPS+ vehicle, LPS + DHLA (30 mg/kg), LPS + DHLA (30 mg/kg) + AAV-control-siRNA, LPS + DHLA (30 mg/kg) + AAV-Nrf2-siRNA. The expression of ERK, Nrf2, HO-1, NLRP3, caspase-1, and IL-1β was detected by Western blot. The test of body weight, OFT, and FST was used to assess the anti-depression effects of DHLA.

### Drug treatment

LPS (*Escherichia coli* 055:B5, Sigma) was solubilized in dimethyl sulfoxide (DMSO) and phosphate-buffered saline (PBS) and administered intraperitoneally (i.p.) at a dosage of 500 μg/kg every 2 days as described previously [20]. DHLA (Sigma) was diluted in DMSO and PBS and administered intraperitoneally (i.p.) daily to a total of 14 injections. The ERK antagonist PD98059 (MedChemExpress, 0.3 mg/kg) was diluted in DMSO and PBS and administered intraperitoneally (i.p.) daily [21]. Flu (Aladdin Reagent Shanghai) was solubilized in sterile distilled water and administered intraperitoneally (i.p.) at a dosage of 10 mg/kg/day [22].

### Hippocampal administration

Adeno-associated virus-mediated small interfering RNA against Nrf2 (AAV-Nrf2-siRNA) or control vector (AAV-Control-siRNA) with an enhanced green fluorescent protein (eGFP) was purchased from Genechem Co., Ltd. (Shanghai, China). The siRNA sequence for Nrf2 is 5′-GTCTTCAGCATGTTACGTGATGAGGATGG-3′ [23]. Hippocampal AAV virus administration was performed as described previously [24]. Briefly, rats were anesthetized with 10% chloral hydrate (0.35 mL/100 g, i.p.) and placed in a stereotaxic apparatus. Rats were infused bilaterally with 1 μL of purified and concentrated AAV virus (1.08 × 10^13^ v.g/mL) into the hippocampus region (coordinates from the bregma: − 3.5 mm posterior, ± 2.3 mm lateral, − 3.0 mm ventral) using an electric microinjection pump (Stoelting, USA). The needle was kept in place for 5 min after infusion and then removed slowly. Subsequently, the incision was closed with interrupted silk sutures, and the animal was placed in a heated cage (35 °C) and monitored carefully.

### FST

FST was performed as described previously. The rats were singly placed in glass cylinders (40-cm height and 28-cm diameter) filled with 30 cm of water (25 ± 1 °C) for 15 min for training, and after 24 h, the animals were placed again in the cylinders for 6 min. The immobile time was recorded during the final 4 min. The immobility was defined as floating with only minimal movements to maintain their head above water.

### Oft

OFT was performed to measure spontaneous activity, as described previously [25]. Briefly, each test rat was placed in the apparatus consisting of a black square 100 cm × 100 cm. The evaluations of every rat were recorded for 5 min, and the rat movements were recorded by a video tracking system (Ethovision XT 11.5). The frequencies of rearing, total distance, and total speed were analyzed using the video tracking system. The apparatus was cleaned with 70% alcohol after each test.

### Flow cytometry

Intracellular oxidative stress was determined by flow cytometry using a ROS assay kit (Jiancheng Biotechnology, Nanjing, China) according to the manufacturer’s protocol [26]. 2,7-Dichlorofluorescein-diacetate (DCFH-DA) was utilized as a sensitive nonfluorescent precursor dye. It permeates the cells and is hydrolyzed by intracellular esterase to the nonfluorescent DCFH, which is rapidly oxidized to the highly fluorescent 2,7-dichlorofluorescein (DCF) in the presence of ROS. The fluorescence intensity of DCF was proportional to the level of intracellular ROS as measured by flow cytometry.

### Western blot analysis

Western blot analysis was performed as described previously [27]. One day after the behavioral tests, the rats were anesthetized with 10% chloral hydrate (3.5 mL/100 g), and the brains were collected. Total protein was prepared from the hippocampus, and the BCA assay was used to analyze the concentration of proteins (BCA Protein Assay, Thermo, 23228). The protein sample was resolved on SDS-PAGE and then transferred to a PVDF membrane. The membranes were probed overnight with the following primary antibodies at 4 °C: Nrf2 (Abcam, ab137550; 1:1000), HO-1 (Abcam, ab13243; 1:1000), ERK (Abcam, ab17942; 1:1000), p-ERK (Abcam, ab50011; 1:1000), NLRP3 (Abcam, ab214185; 1:1000), caspase-1 (Abcam, ab179515; 1:1000), and IL-1β (Abcam, ab9722; 1:1000). Subsequently, the membranes were washed with TBST (3 times for 5 min each) and incubated with goat anti-rabbit IgG (1:2000; Abcam, ab205718) and goat anti-mouse IgG (1:2000; Abcam, ab205719), as appropriate, for 1 h at room temperature. The immunoreactive bands were developed by chemiluminescence using Chemi Doc XRS System (Bio-rad, USA), and the intensities were normalized to that of GAPDH (1:10000; Servicebio, GB11002) used as an internal standard and quantified using ImageJ software.

### Immunofluorescence

Immunofluorescence staining was conducted as described previously [28]. A series of 30 μm slices were blocked in 2% BSA and incubated overnight with the primary antibody at 4 °C. Primary antibodies: anti-HO-1 (Abcam, ab13243; 1:1000) and anti-IBA-1(Wako, 01919741; 1:1000) were used as primary antibodies. The secondary antibody was probed on the slide for 1 h in the dark. Subsequently, the sections were washed three times with PBS before the images were acquired using a Nikon upright fluorescence microscope.

### Statistical analysis

Prism software (GraphPad Prism 8.0, CA, USA) was used for all the analyses. The data are presented as mean ± standard error of the mean (SEM). The normality of the distribution was assessed by the Shapiro-Wilk test. Statistical comparisons were made using one-way analysis of variance (ANOVA), followed by Tukey’s post hoc test. Statistical significance was indicated by *p* value < 0.05.

## Results

### DHLA treatment reversed the LPS-induced sickness behavior

Bodyweight gain and behavioral tests, including OFT and FST, were performed to investigate the effects of DHLA on LPS-induced sickness behavior in rats.

**Fig. 2:**
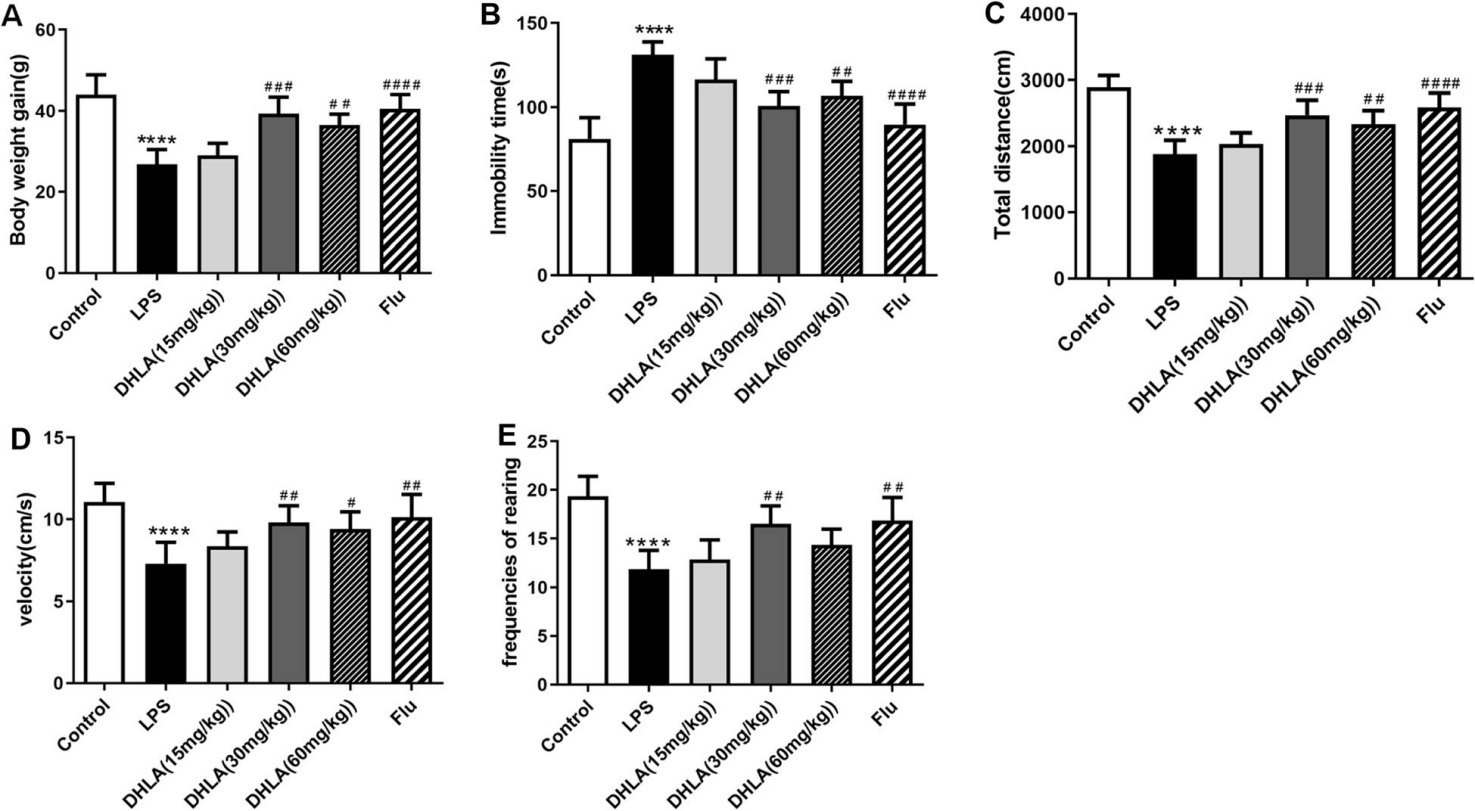
Effect of different doses of DHLA on treatment of sickness behavior rats. **a** Effect of DHLA and Flu on body weight changes. **b**–**e** Depression-like behaviors were assessed by forced swimming test (**b**) and open field test (**c**–**e**). The data were expressed as means ± SEM (*n* = 6). ****P < 0.0001 versus the control group. ^#^*P* < 0.05; ^##^*P* < 0.01; ^###^*P* < 0.001; ^####^*P* < 0.0001 versus the LPS group. The Shapiro-Wilk test results showed that all the data are normally distributed (*p* > 0.05)

As shown in Fig. 2a, rats exposed to LPS showed less body weight gain as compared to the control group (*F*(5,30) = 19.83, *p* < 0.0001). However, treatment with DHLA (30 mg/kg, *p* < 0.001; 60 mg/kg, *p* < 0.01) and Flu (*p* < 0.0001) improved the body weight gain as compared to the LPS group.

The FST is mainly used to measure the depression-like behavior. As shown in Fig. 2b, rats exposed to LPS showed more immobility time in FST as compared to the control group (*F*(5,30) = 17.49, *p* < 0.0001), whereas compared to the LPS group, DHLA (30 mg/kg, *p* < 0.001; 60 mg/kg, *p* < 0.01) and Flu (*p* < 0.0001) treatment markedly decreased the immobility time in FST.

The performance of rats in OFT is shown in Fig. 2c–e. The total distance, total velocity, and rearing frequencies were significantly decreased in the LPS group as compared to the control group (total distance (*F*(5,30) = 19.93, *p* < 0.0001); velocity (*F*(5,30) = 8.132, *p* < 0.0001); rearing frequencies (*F*(5,30) = 11.66, *p* < 0.0001). Compared to the LPS group, the total distance in the DHLA (30 mg/kg, *p* < 0.001; 60 mg/kg, *p* < 0.01) and Flu (*p* < 0.0001) groups was significantly increased, the total velocity in the DHLA (30 mg/kg, *p* < 0.01; 60 mg/kg, *p* < 0.05) and Flu (*p* < 0.01) groups were significantly increased, and the rearing frequencies in the DHLA (30 mg/kg, *p* < 0.01) and Flu (*p* < 0.01) groups were significantly increased.

Since DHLA (30 mg/kg) was superior to the other doses in measurements (Fig. 2a–e), we selected 30 mg/kg as the optimal dose of DHLA and used it in the following experiments.

### DHLA inhibited the activation of microglia induced by LPS

To measure the number of microglia cells, immunofluorescence staining was performed by using the microglia-specific marker, IBA-1. As shown in Additional file 3: Fig. S3 A-B, rats exposed to LPS showed more number microglia as compared to the control group (*F*(4,25) = 22.16, *p* < 0.001). However, treatment with DHLA (*p* < 0.01) decreased the microglia cell number as compared to the LPS group.

### DHLA reversed the LPS-induced sickness behavior through ERK/Nrf2/HO-1/ROS/NLRP3-dependent inflammation pathway

Western blot and immunofluorescence staining were used to test the expression of ERK/Nrf2/HO-1/ROS/NLRP3 signaling pathway in response to DHLA against LPS-induced sickness behavior in rats.

**Fig. 3:**
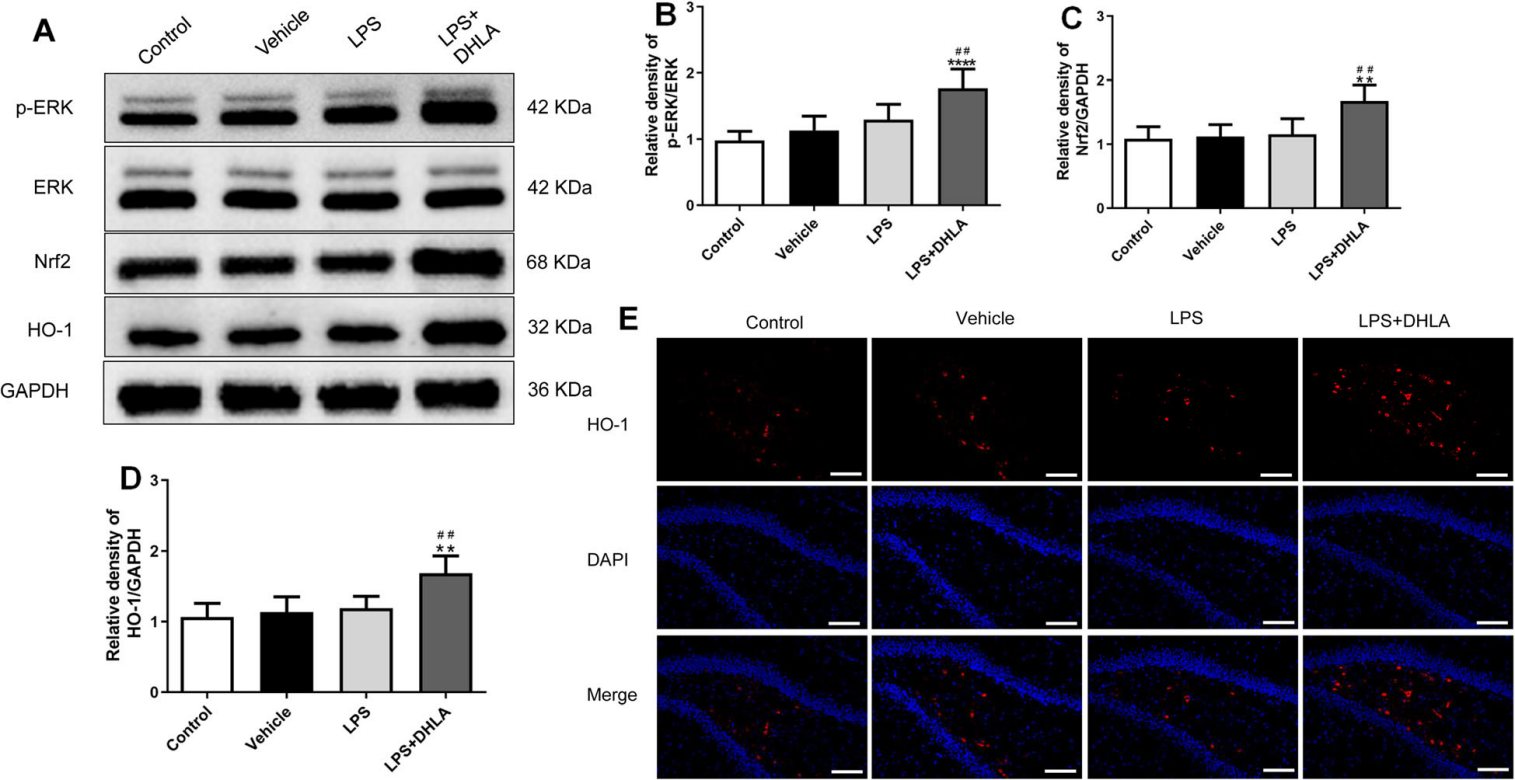
Effect of DHLA on ERK/Nrf2/HO-1 signaling pathway in rats. **a** Representative Western blot bands in the hippocampal region. **b**–**d** Statistical graphs of relative protein expression of p-ERK/ERK (**b**), Nrf2 (**c**), and HO-1 (**d**). The data were expressed as means ± SEM (*n* = 6). **e** Representative images of immunofluorescence assays of HO-1 in the hippocampal. Scale bars represent 50 μm. The data were expressed as means ± SEM (*n* = 3). **P < 0.01; ****P < 0.0001 versus the control group. ^##^*P* < 0.01 versus the LPS group. The Shapiro-Wilk test results showed that all the data are normally distributed (*p* > 0.05)

**Fig. 4:**
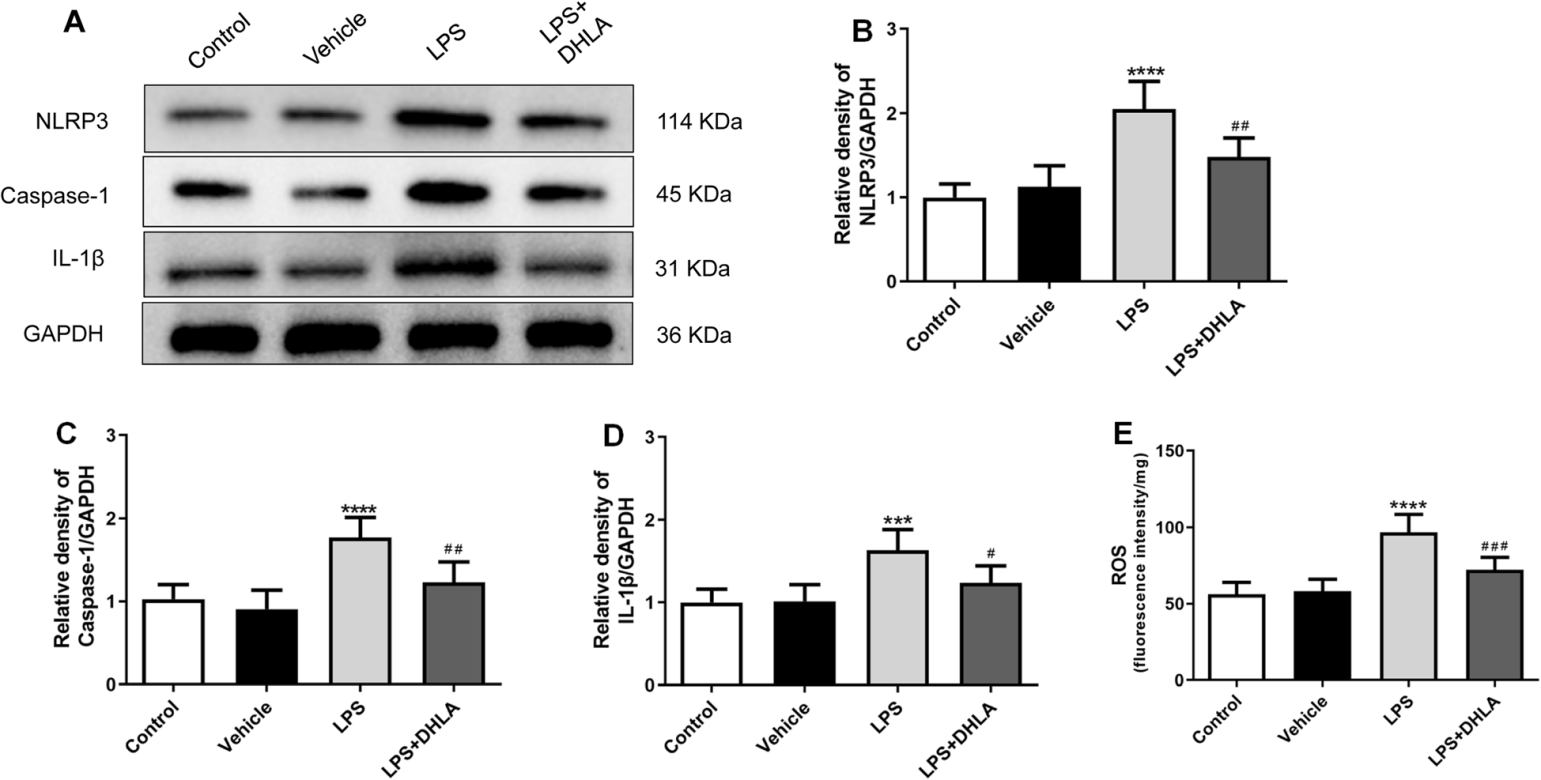
Effect of DHLA on ROS/NLRP3/caspase-1/IL-1β signaling pathway in rats. **a** Representative Western blot bands in the hippocampal region. **b**–**d** Statistical graphs of relative protein expression of NLRP3 (**b**), caspase-1 (**c**), and IL-1β (**d**). **e** ROS expression in the hippocampal. The data were expressed as means ± SEM (*n* = 6). ***P < 0.001; ****P < 0.0001 versus the control group. ^#^*P* < 0.05; ^##^*P* < 0.01; ^###^*P* < 0.001 versus the LPS group. The Shapiro-Wilk test results showed that all the data are normally distributed (*p* > 0.05)

As shown in Fig. 3a–d, a statistically significant difference between groups for p-ERK (*F*(3,20) = 13.97, *p* < 0.0001), Nrf2 (*F*(3,20) = 9.48, *p* < 0.0001), and HO-1 (*F*(3,20) = 10.90, *p* < 0.0001) was determined by one-way ANOVA. Tukey’s post hoc analysis revealed that expression levels of p-ERK (*p* < 0.01), Nrf2 (*p* < 0.01), and HO-1 (*p* < 0.01) were significantly higher in DHLA-treated rats as compared to the LPS groups. Immunofluorescence staining revealed that the expression of HO-1 in the DHLA group was increased than that in the control group (Fig. 3e). As shown in Fig. 4a–d, a statistically significant difference in NLRP3 (*F*(3,20) = 21.62, *p* < 0.0001), caspase-1 (*F*(3,20) = 17.37, *p* < 0.0001), and IL-1β(*F*(3,20) = 11.93, *p* < 0.0001) between the groups as determined by one-way ANOVA. Tukey’s post hoc analysis revealed that the expression levels of NLRP3 (*p* < 0.0001), caspase-1 (*p* < 0.0001), and IL-1β (*p* < 0.001) were significantly higher in LPS rats as compared to the control groups. However, these effects were significantly reversed by the treatment with DHLA (NLRP3, *p* < 0.01; caspase-1, *p* < 0.01; IL-1β, *p* < 0.05).

To verify the effect of DHLA alone on ERK/Nrf2/HO-1/NLRP3 signaling pathway, we designed another four groups. As shown in Additional file 2: Fig. S2, a statistically significant difference between groups for p-ERK (*F*(3,20) = 30.09, *p* < 0.0001), Nrf2 (*F*(3,20) = 10.57, *p* < 0.0001), HO-1 (*F*(3,20) = 12.03, *p* < 0.0001), NLRP3 (*F*(3,20) = 28.55, *p* < 0.0001), and IL-1β (*F*(3,20) = 16.58, *p* < 0.0001) was determined by one-way ANOVA. Tukey’s post hoc analysis revealed that expression levels of p-ERK (*p* < 0.01), Nrf2 (*p* < 0.01), and HO-1 (*p* < 0.01) were significantly higher in DHLA alone group as compared to the LPS groups, and the expression levels of NLRP3 (*p* < 0.01) and IL-1β (*p* < 0.05) were significantly lower in DHLA alone group as compared to the LPS groups.

The concentration of intracellular ROS was evaluated by the changes in DCF fluorescence intensity using flow cytometry. As shown in Fig. 4e, the ROS expression was distinctly higher in the LPS rats as compared to the control group (*F*(3,20) = 25.80, *p* < 0.0001). Conversely, the intracellular ROS level induced by LPS was markedly reversed by DHLA (*p* < 0.001).

Bodyweight gain and behavioral tests, including OFT and FST, were performed to further investigate the effects of DHLA on LPS-induced sickness behavior in rats. As shown in Additional file 1: Fig. S1A, rats exposed to LPS showed less body weight gain than the control group (*F*(5,30) = 20.84, *p* < 0.0001). However, treatment with DHLA (30 mg/kg, *p* < 0.01) improved the bodyweight gain as compared to the LPS group. Additional file 1: Fig. S1B showed that rats exposed to LPS showed more immobility time in FST as compared to the control group (*F*(5,30) = 27.11, *p* < 0.0001). On the other hand, compared to the LPS group, DHLA (30 mg/kg, *p* < 0.001) treatment markedly decreased the immobility time in FST. As shown in Additional file 1: Fig. S1C–E, the total distance, total velocity, and rearing frequencies were significantly decreased in the LPS group as compared to the control group (total distance (*F*(5,30) = 30.48, *p* < 0.0001); velocity (*F*(5,30) = 12.16, *p* < 0.0001); rearing frequencies (*F*(5,30) = 15.22, *p* < 0.0001). Compared to the LPS group, the total distance, total velocity, and rearing frequencies in the DHLA (30 mg/kg, all *p* < 0.01) group were significantly increased.

### Blockade of ERK abolished the preventive effect and anti-inflammation effect of DHLA

Previous studies have suggested that the ERK pathway is related to the regulation of Nrf2 [29]. PD98059 (an ERK pathway inhibitor) was administered in LPS-induced sickness behavior rats 1 h before the administration of DHLA to investigate whether DHLA upregulated the expression of Nrf2/HO-1 in LPS-induced rats via the ERK pathway.

**Fig. 5:**
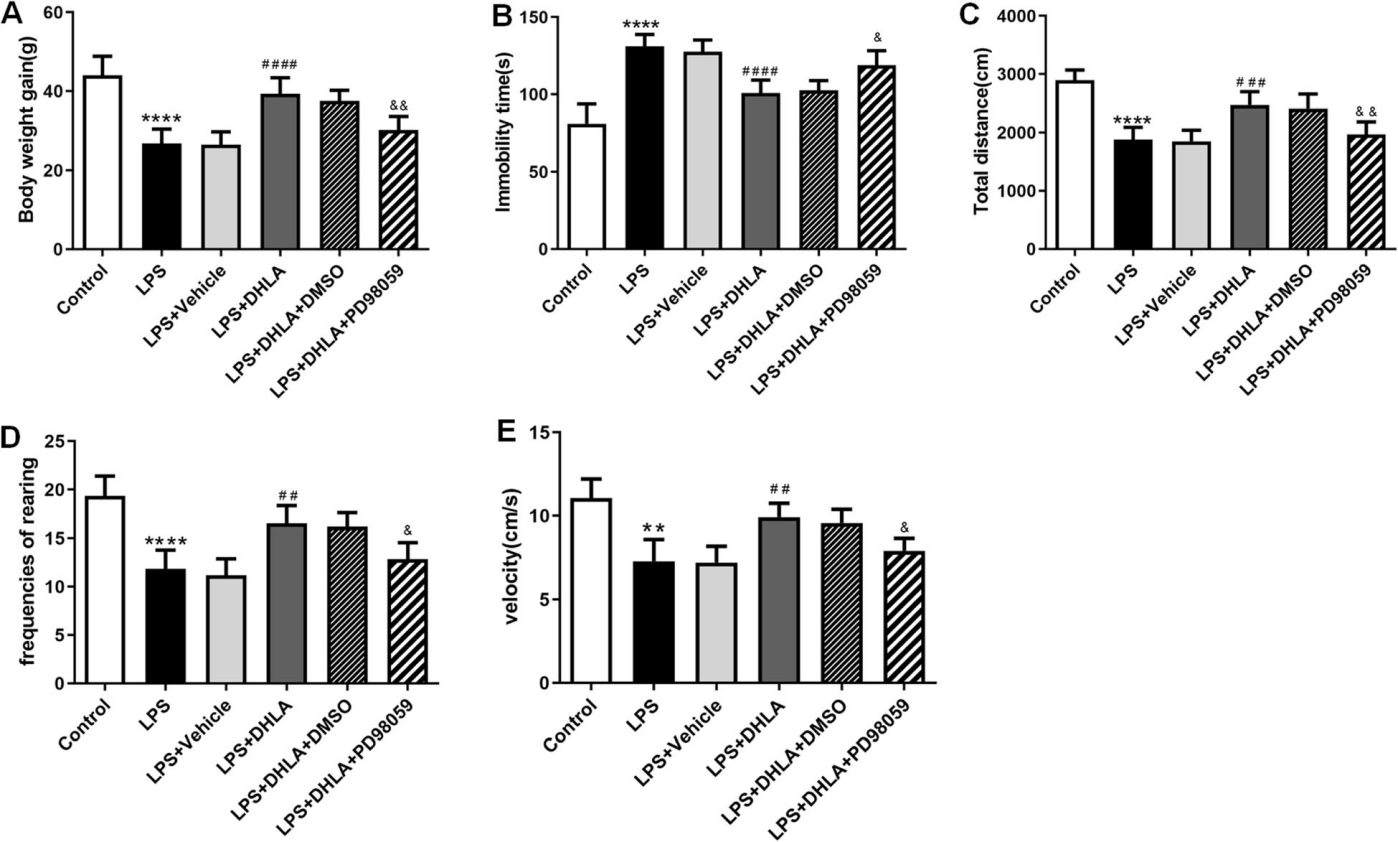
Blockade of ERK abolished the preventive effect of DHLA. **a** Effect of DHLA on body weight changes. **b**–**e** Depression-like behaviors were assessed by forced swimming test (**b**) and open field test (**c**–**e**). The data were expressed as mean ± SEM (*n* = 6). **P < 0.01; ****P < 0.0001 versus the control group. ^##^*P* < 0.01; ^###^*P* < 0.001; ^####^*P* < 0.0001 versus the LPS group. ^&^*P* < 0.05; ^&&^*P* < 0.01 versus the LPS + DHLA group. The Shapiro-Wilk test results showed that all the data are normally distributed (*p* > 0.05)

As shown in Fig. 5a, rats exposed to DHLA showed higher body weight gain as compared to the LPS group (*F*(5,30) = 22.61, *p* < 0.0001). However, treatment with PD98059 (*p* < 0.01) decreased body weight gain than the DHLA group.

As shown in Fig. 5b–e, the DHLA administration greatly attenuated the depression-like behavior in LPS-induced rats. As shown in Fig. 5b, rats exposed to DHLA showed less immobility time in FST as compared to the LPS group (*F*(5,30) = 26.79, *p* < 0.0001), whereas compared to the DHLA group, the PD98059 (*p* < 0.05) treatment markedly increased the immobility time in FST. As shown in Fig. 5c–e, the total distance, rearing frequencies, and total velocity were significantly increased in the DHLA group as compared to the LPS group (total distance *F*(5,30) = 22.78, *p* < 0.0001; rearing frequencies *F*(5,30) = 18.71, *p* < 0.0001; velocity *F*(5,30) = 15.17, *p* < 0.0001). However, compared to the DHLA group, the total distance in the PD98059 (*p* < 0.01) group and rearing frequencies and total velocity in the PD98059 (both *p* < 0.05) group were significantly decreased. These data indicated that the preventive effect of DHLA in the LPS-induced rats was blocked by PD98059.

**Fig. 6:**
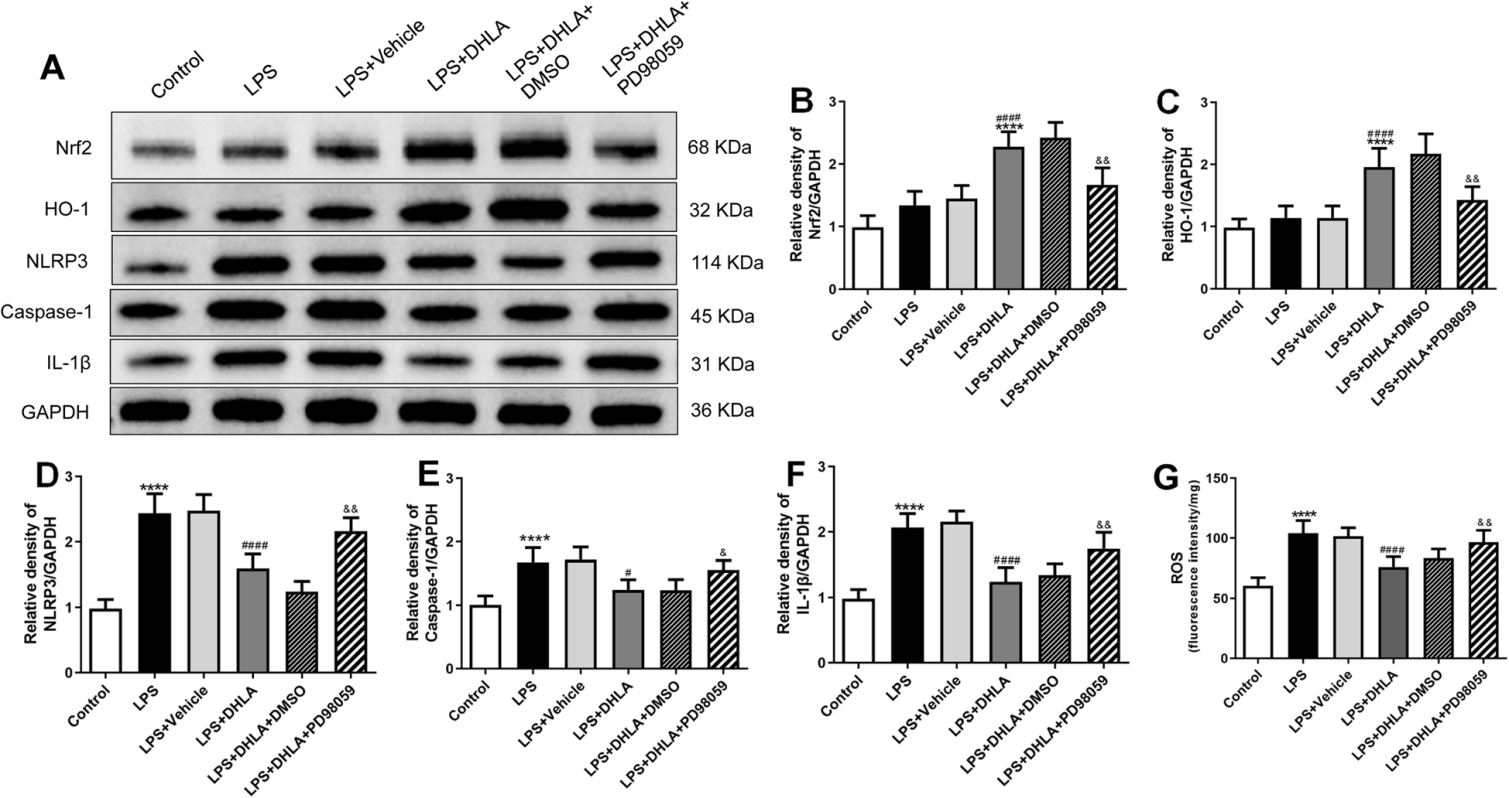
Blockade of ERK abolished the anti-Inflammation effect of DHLA. **a** Representative Western blot bands in the hippocampal region. **b**–**f** Statistical graphs of relative protein expression of Nrf2 (**b**), HO-1 (**c**), NLRP3 (**d**), caspase-1 (**e**), and IL-1β (**f**). **g** ROS expression in the hippocampal. The data were expressed as means ± SEM (*n* = 6). ****P < 0.0001 versus the control group. ^#^*P* < 0.05; ^####^*P* < 0.0001 versus the LPS group. ^&^*P* < 0.05; ^&&^*P* < 0.01 versus the LPS + DHLA group. The Shapiro-Wilk test results showed that all the data are normally distributed (*p* > 0.05)

As shown in Fig. 6a–f, the results showed a statistically significant difference between the study groups as determined by one-way ANOVA with respect to Nrf2 (*F*(5,30) = 35.07, *p* < 0.0001), HO-1 (*F*(5,30) = 25.19, *p* < 0.0001), NLRP3(*F*(5,30) = 50.79, *p* < 0.0001), caspase-1 (*F*(5,30) = 15.64, *p* < 0.0001), and IL-1β (*F*(5,30) = 34.71, *p* < 0.0001). Tukey’s post hoc analysis revealed that expression levels of Nrf2 (*p* < 0.0001) and HO-1 (*p* < 0.0001) were significantly higher in DHLA rats as compared to the LPS groups. In addition, the expression of NLRP3 (*p* < 0.0001), caspase-1 (*p* < 0.01), and IL-1β (*p* < 0.0001) was significantly lower in DHLA rats as compared to the LPS groups. However, inhibition of ERK with PD98059 abolished the effects of DHLA, which led to decreased Nrf2 (*p* < 0.01) and HO-1 (*p* < 0.01), while increased in the inflammation-related proteins, such as NLRP3 (*p* < 0.01), caspase-1 (*p* < 0.05), and IL-1β (*p* < 0.01) as compared to the DHLA group.

As shown in Fig. 6g, the ROS expression was distinctly lower in the DHLA group as compared to the LPS group (*F*(5,30) = 23.09, *p* < 0.0001). Conversely, the ROS level induced by DHLA was markedly ameliorated by PD98059 (*p* < 0.01).

### Blockade of Nrf2 abolished the preventive effect and anti-inflammation effect of DHLA

Bodyweight gain and behavioral tests showed that AAV-Nrf2-siRNA completely abolished the treatment effects of DHLA in the LPS-induced sickness behavior rats.

**Fig. 7:**
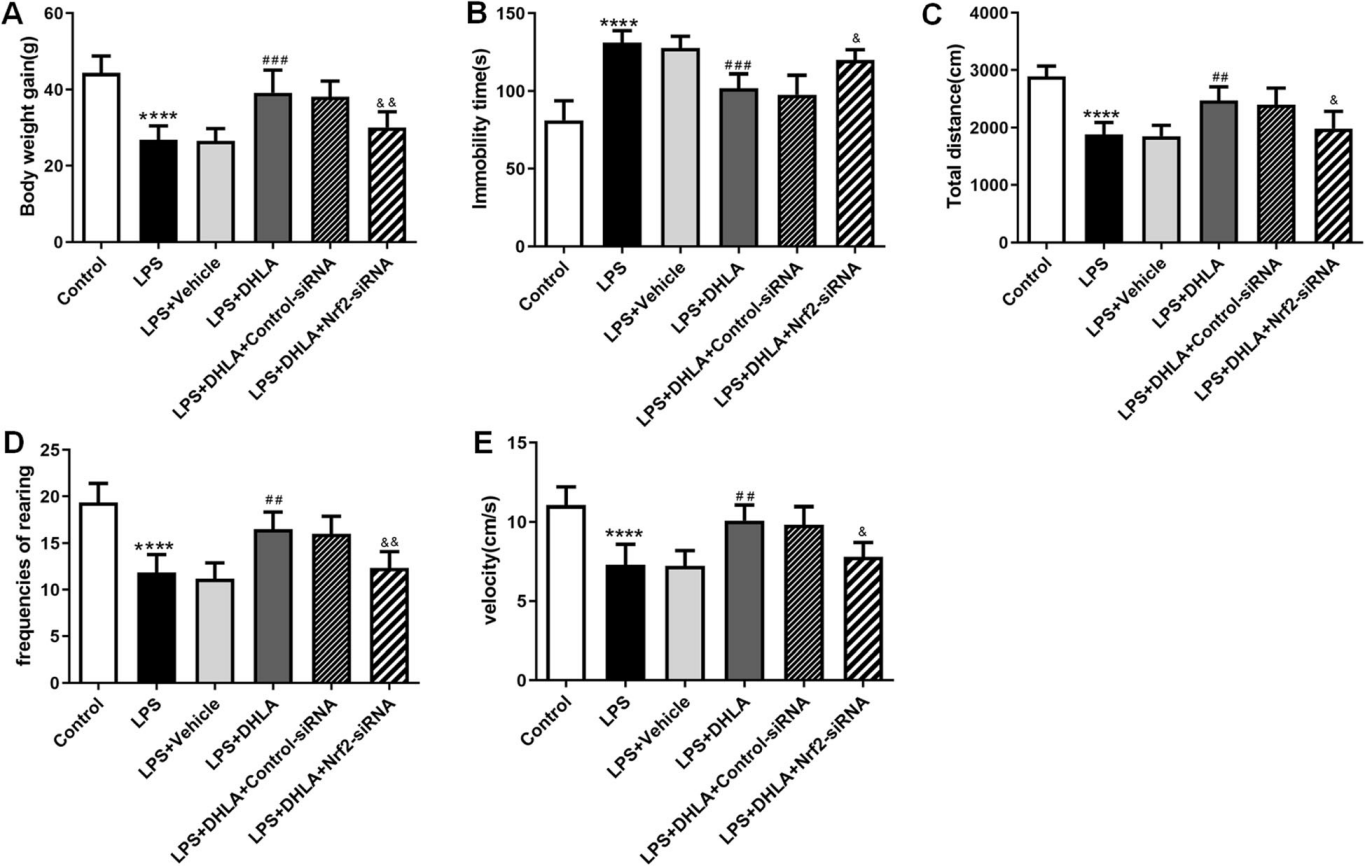
Blockade of Nrf2 abolished the preventive effect of DHLA. **a** Effect of DHLA on body weight changes. **b**–**e** Depression-like behavior was assessed by forced swimming test (**b**) and open field test (**c**–**e**). The data were expressed as means ± SEM (*n* = 6). ****P < 0.0001 versus the control group. ^##^*P* < 0.01; ^###^*P* < 0.001 versus the LPS group. ^&^*P* < 0.05; ^&&^*P* < 0.01 versus the LPS + DHLA group. The Shapiro-Wilk test results showed that all the data are normally distributed (*p* > 0.05)

As shown in Fig. 7a, rats exposed to DHLA showed higher body weight gain as compared to the LPS group (*F*(5,30) = 17.46, *p* < 0.0001). However, treatment with AAV-Nrf2-siRNA (*p* < 0.05) decreased body weight gain as compared to the DHLA group.

As shown in Fig. 7b–e, the DHLA administration greatly attenuated the sickness behaviors observed in LPS-induced rats. As shown in Fig. 7b, rats exposed to DHLA showed less immobility time in FST as compared to the LPS group (*F*(5,30) = 24.1, *p* < 0.0001). On the other hand, compared to the DHLA group, the AAV-Nrf2-siRNA (*p* < 0.05) treatment markedly increased the immobility time in FST. As shown in Fig. 7c–e, the total distance, rearing frequencies, and total velocity were significantly increased in the DHLA group that in the LPS group (total distance *F*(5,30) = 17.84, *p* < 0.0001; rearing frequencies *F*(5,30) = 17.87, *p* < 0.0001; velocity *F*(5,30) = 13.86, *p* < 0.0001). However, compared to the DHLA group, the total distance, the rearing frequencies, and total velocity in the DHLA (*p* < 0.05, *p* < 0.01, *p* < 0.05, respectively) group were significantly decreased. These data indicated that the preventive effect of DHLA in LPS-induced rats was blocked by AAV-Nrf2-siRNA.

**Fig. 8:**
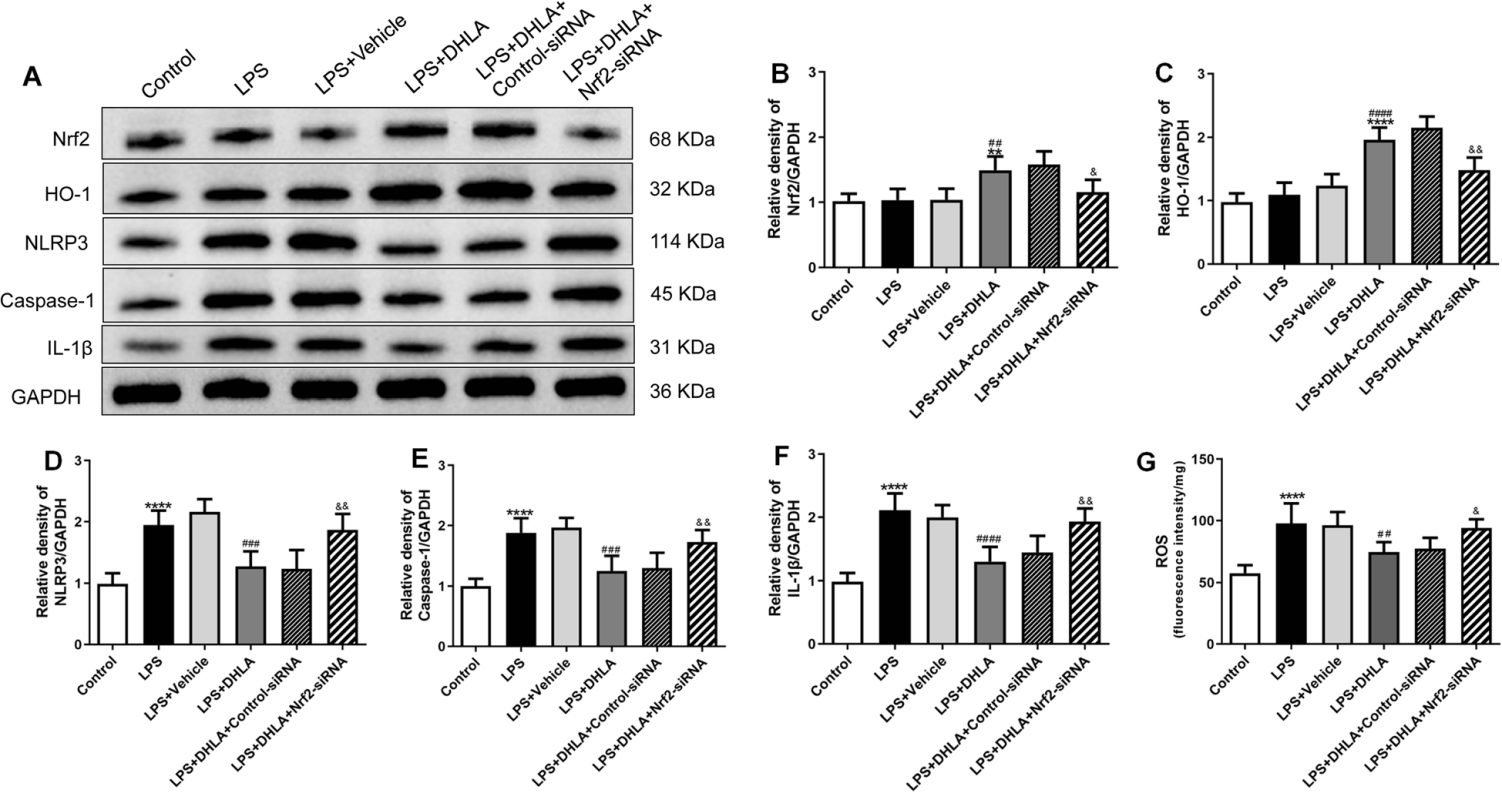
Blockade of Nrf2 abolished the anti-Inflammation effect of DHLA. **a** Representative Western blot bands in the hippocampal region. **b**–**f** Statistical graphs of relative protein expression of Nrf2 (**b**), HO-1 (**c**), NLRP3 (**d**), caspase-1 (**e**), and IL-1β (**f**). **g** ROS expression in the hippocampal. The data were expressed as means ± SEM (*n* = 6). **P < 0.0001; ****P < 0.0001 versus the control group. ^##^*P* < 0.01; ^###^*P* < 0.001; ^####^*P* < 0.0001 versus the LPS group. ^&^*P* < 0.05; ^&&^*P* < 0.01 versus the LPS + DHLA group. The Shapiro-Wilk test results showed that all the data are normally distributed (*p* > 0.05)

As shown in Fig. 8a–e, the results showed a statistically significant difference between the study groups as determined by one-way ANOVA regarding Nrf2 (*F*(5,30) = 11.77, *p* < 0.0001), HO-1 (*F*(5,30) = 41.88, *p* < 0.0001), NLRP3 (*F*(5,30) = 23.01, *p* < 0.0001), caspase-1 (*F*(5,30) = 20.75, *p* < 0.0001), and IL-1β (*F*(5,30) = 25.14, *p* < 0.0001). Tukey’s post hoc analysis revealed that the expression levels of Nrf2 (*p* < 0.01) and HO-1 (*p* < 0.0001) were significantly higher in DHLA rats as compared to that in the LPS groups. Moreover, the expression of NLRP3 (*p* < 0.001), caspase-1 (*p* < 0.001), and IL-1β (*p* < 0.0001) was significantly lower in DHLA rats as compared to the LPS groups. However, inhibition of Nrf2 with AAV-Nrf2-siRNA abolished the effects of DHLA, which led to a decrease in Nrf2 (*p* < 0.05) and HO-1 (*p* < 0.01) expression and increase in the expression of inflammation-related proteins NLRP3 (*p* < 0.01), caspase-1 (*p* < 0.01), and IL-1β (*p* < 0.001) as compared to the DHLA group.

As shown in Fig. 8g, the results showed that the ROS expression was distinctly lower in the DHLA group as compared to the LPS group (*F*(5,30) = 14.89, *p* < 0.0001). In contrast, the ROS level induced by DHLA was markedly ameliorated by AAV-Nrf2-siRNA (*p* < 0.05).

## Discussion

The present study revealed that the sickness behaviors induced by LPS in rats are reversed by DHLA treatment with the anti-depressant Flu and is associated with the upregulation of Nrf2 and HO-1. The inhibition of ERK abolished the preventive effect of DHLA, which was related to the low expression of Nrf2 and HO-1 and upregulation of NLRP3, caspase-1, and IL-1β. Moreover, the knockdown of Nrf2 using Nrf2 siRNA abolished the effect of the DHLA treatment effect similarly, which was associated with a decrease in HO-1 and upregulation of NLRP3, caspase-1, and IL-1β. Taken together, our findings suggested that DHLA protects against LPS-induced behavioral deficits and neuroinflammation through ERK/Nrf2/HO-1/ROS/NLRP3 signaling pathway in rats.

Lipoic acid is a natural lipophilic antioxidant that has beneficial effects on heart diseases, diabetes, and neuroinflammatory disorders [30–33]. Lipoic acid occurs in two forms: α-lipoic acid and DHLA [34]. DHLA is the reduced form of lipoic acid with a more specific and useful outcome than α-lipoic acid [35]. DHLA is considered to have more antioxidant properties than LA, because DHLA has the properties of endogenous antioxidants regeneration and repairing oxidative damage [36]. Moreover, the neuroprotective effects of DHLA have been shown in several central nervous system diseases, such as ischemic stroke, traumatic brain injury. Recently, DHLA was indicated as an effective treatment for subarachnoid hemorrhage [19]. However, no study has been published, wherein DHLA was used as a therapeutic method for depression. To the best of our knowledge, this is the first study evaluating the preventive effect of DHLA on sickness behaviors in rats.

Herein, we first evaluated the effect of three different dosages of DHLA on LPS-induced sickness behavior in rats. In the current study, 30 mg/kg DHLA is the most effective dose for the treatment of sickness behaviors. In line with our results, a previous study has suggested that 30 mg/kg DHLA contributes to the neuroprotective effects [19]. Thus, this dose may be optimal for the treatment of nervous system disease.

LPS is a component of the outer cell wall of Gram-negative bacteria and is widely used to induce inflammation in the brain. Nrf2 is a major transcription factor that regulates cell antioxidant response. Nrf2 is transported to the nucleus under oxidative stress and binds to the antioxidant response elements (ARE) to regulate the expression of antioxidant enzymes such as HO-1 [37]. In the previous study, the expression of the Nrf2-systerm (Nrf2 and HO-1) in the rat brain was still increased 3 days after the LPS systemic injection, decreased by 7 days, and then gradually normalized after LPS [38]. Our results showed that the contents of Nrf2 and HO-1 were nearly normalized in the hippocampus of rats after 14 days of LPS-induced. The temporal shift of Nrf2-systerm is likely caused by a prolonged acute phase of inflammation as a result of the higher dose of LPS. Consecutively, we found that DHLA treatment increased the expression of Nrf2 and HO-1, which was abrogated when Nrf2 was downregulated by AAV-Nrf2-siRNA.

Previous studies indicated that upregulated HO-1 expression exerts a protective effect against increased levels of ROS [39]. In the present study, the ROS level increased significantly in the LPS-induced groups as compared to the control groups but was restored in the DHLA treatment group.

Therefore, we speculated that DHLA regulates the ROS expression by activating the Nrf2/HO-1 signaling pathway. However, the mechanisms underlying the DHLA-mediated and Nrf2 activation after LPS-induced sickness behavior have not yet been elucidated. Previous studies have shown that Nrf2 activation is regulated by the MAPK/ERK pathway [40]. We postulated that treatment with DHLA might regulate Nrf2 expression via the ERK pathway in rats. To further confirm our hypothesis, we used an inhibitor of ERK to test the expression of Nrf2 after DHLA treatment. Previous studies have shown that LPS can significantly increase phosphorylation levels of ERK in the hippocampus of mice [41, 42]. At the same time, one study demonstrated that LPS did not affect ERK phosphorylation in the hippocampus of mice [43]. In our study, we observed that LPS did not affect the phosphorylation of ERK in the hippocampus. The different expression of p-ERK induced by LPS may be related to the dose of LPS and stimulation time of LPS. Then, we found that DHLA promoted the expression of ERK and Nrf2. Strikingly, the expression of Nrf2 could be inhibited by the ERK inhibitor PD98059. Thus, we speculated that DHLA activates Nrf2 through the ERK signaling pathway. These findings indicated that DHLA improves the sickness behaviors induced by LPS in rats, primarily through the ERK/Nrf2/HO-1/ROS pathway.

In recent years, many studies have shown that the pathogenesis and progression of depression are related to the overreaction of inflammation and immune responses [4, 44]. The inflammasome is an inducer of immune response with the function of identifying and targeting multiple pathogens [45]. Hitherto, several forms of inflammasomes have been reported, including NLRP1, NLRP2, NLRP3, and NLRC4 inflammasomes [46]. Among these, NLRP3 inflammasome is involved in the onset and progression of several diseases [47]. It is composed of NLRP3 protein, adaptor protein apoptosis-related speckles (ASC), and procaspase-1 [48, 49]. The activation of NLRP3 inflammasome triggers the transformation of procaspase-1 to caspase-1 and catalyzes the exudation of mature IL-1β and IL-18 from pro-IL-1β and pro-IL-18, causing an inflammatory response [50, 51]. Previous studies showed that ROS, especially from mitochondria, activated the NLRP3 inflammasome [52, 53]. The current findings proposed that the expression of ROS, NLRP3, caspase-1, and IL-1β in the LPS group was increased as compared to that in the control groups; however, after the administration of DHLA, the level of NLRP3, caspase-1, and IL-1β decreased markedly.

Microglia are the main immune cells in the CNS and are involved in neuroprotection and immunosurveillance through the regulation of various pro-inflammatory cytokines [54]. Some studies have shown that microglia is activated in neuroinflammation, contributing to the development of depression [55, 56]. Excessive activation of microglia promotes the production of pro-inflammatory factors, such as IL-1β, and damages synaptic function. Our results showed that the number of microglia was significantly increased in the LPS-induced groups. However, DHLA evidently inhibited the increase of microglial cells. Therefore, we speculated that inhibition of microglia activation may be related to the anti-inflammatory effect of DHLA.

Furthermore, these results supported DHLA treatment to attenuate the oxidative stress-related neuroinflammation and microglial activation for LPS-induced sickness behavior. Nevertheless, our study is not without limitations, first, the downstream mechanism of Nrf2 to NLRP3 is complex; hence, we targeted only one portion of the pathway. Thus, the probability of other factors involved in DHLA treatment needs further investigation. Second, the protective effects of DHLA might be observed in other cell types such as neurons or astrocytes, and hence, these factors also need to be explored further. Third, in our study, we used LPS-injected rats as our animal model, and this model is widely used as a sickness behavior model and seems to be for a limited condition of depression.

## Conclusions

In conclusion, our study suggests that DHLA has a preventive effect on sickness behaviors in rats with LPS-induced, and these effects are exerted via regulation of Nrf2/HO-1/ROS/NLRP3 signaling pathway and microglial activation. These results provide a theoretical basis for DHLA as a promising method in the treatment of depression.

## Supplementary information

**Additional file 1: Figure S1.** Effect of optimal dose of DHLA on treatment of sickness behavior rats. **a** Effect of DHLA on body weight changes. **b-e** Depression-like behavior was assessed by forced swimming test (**b**) and open field test (**c, d, e**). The data were expressed as means ± SEM (n=6). ****P < 0.0001, versus the control group. ^##^*P* < 0.01; ^###^*P* < 0.001 versus the LPS group. The Shapiro-Wilk test results showed that all the data are normally distributed (*p* > 0.05).

**Additional file 2: Figure S2.** Effect of DHLA on ERK/Nrf2/HO-1/NLRP3/IL-1β signaling pathway in rats. **a-b** Representative Western blot bands in the hippocampal region. **c-g** Statistical graphs of relative protein expression of p-ERK/ERK (**c**), Nrf2 (**d**), HO-1 (**e**), NLRP3 (**f**), IL-1β (**g**). The data were expressed as means ± SEM (n=6). **P < 0.01; ***P < 0.001; ****P < 0.0001, versus the control group. ^#^*P* < 0.05; ^##^*P* < 0.01, versus the LPS group. The Shapiro-Wilk test results showed that all the data are normally distributed (*p* > 0.05).

**Additional file 3: Figure S3.** Effect of optimal dose of DHLA prevents LPS-induced increase of the microglial number. **a** Representative images of immunofluorescence assays of Iba1 in the hippocampus. Six micrographs from three rats per group were analyzed. **b** DHLA blocked the increased Iba-1 signal intensity induced by LPS. Scale bars represent 50 μm. The data were expressed as means ± SEM (n=6). ***P < 0.001, versus the control group. ^##^*P* < 0.01 versus the LPS group. The Shapiro-Wilk test results showed that all the data are normally distributed (*p* > 0.05).

## Data Availability

All the necessary data are included within the article. Further data will be shared by request.

## Abbreviations

DHLA: Dihydrolipoic acid
FST: Forced swim test
HO-1: Heme oxygenase-1
LPS: Lipopolysaccharide
NLRP3: Nod-like receptor pyrin containing 3 inflammasome
Nrf2: Nuclear factor erythroid 2-like
OFT: Open field test
ROS: Reactive oxygen species
