# Supplementary figures and images for: Dihydrolipoic acid protects against lipopolysaccharide-induced behavioral deficits and neuroinflammation via regulation of Nrf2/HO-1/NLRP3 signaling in rat
Source: J Neuroinflammation. 2020 May 25;17:166. doi: 10.1186/s12974-020-01836-y (PMC7249417; doi:10.1186/s12974-020-01836-y)

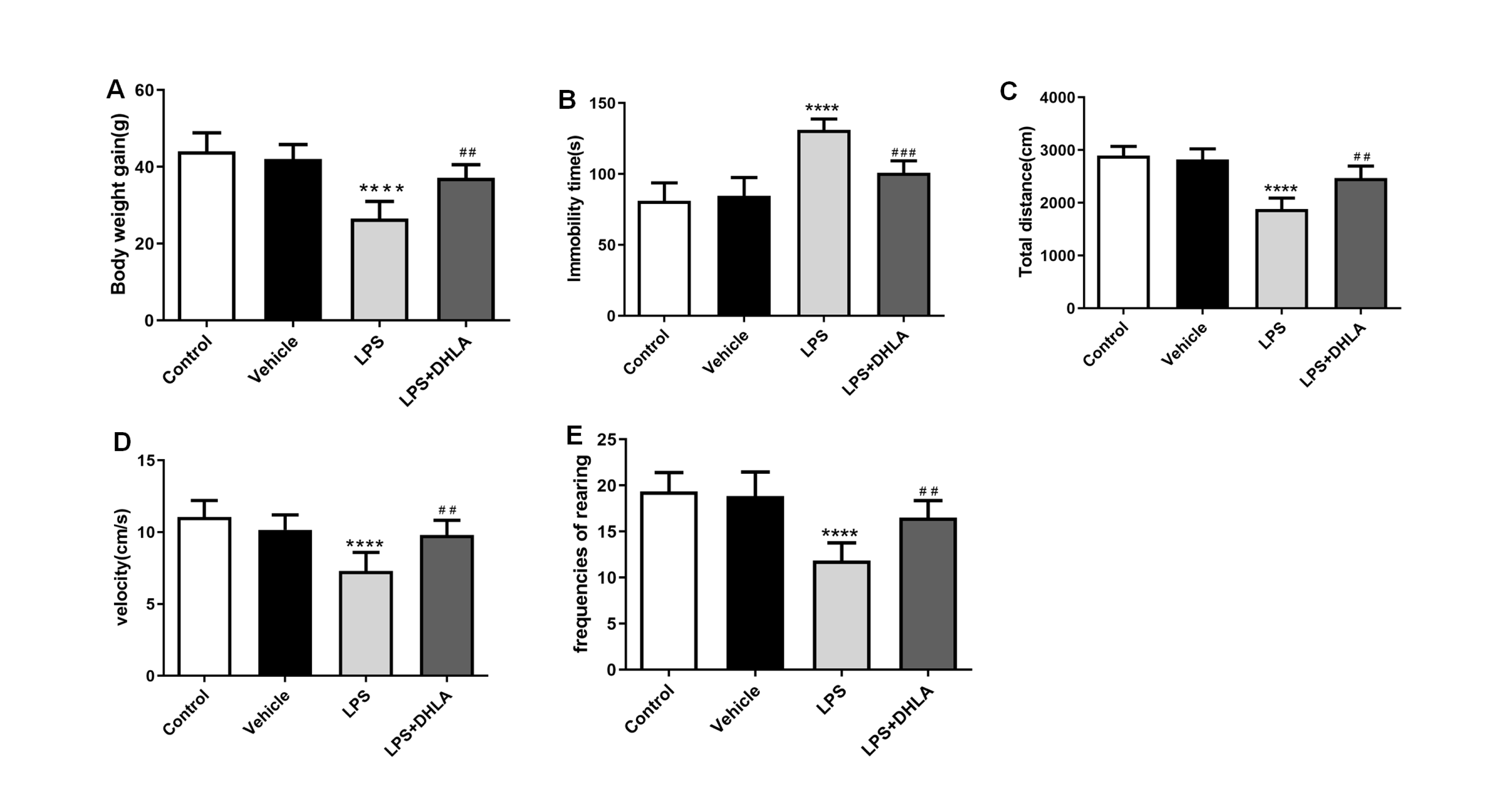

Supplement: Supplementary file 1 — Additional file 1: Figure S1. Effect of optimal dose of DHLA on treatment of sickness behavior rats. a Effect of DHLA on body weight changes. b-e Depression-like behavior was assessed by forced swimming test (b) and open field test (c, d, e). The data were expressed as means ± SEM (n=6). ****P < 0.0001, versus the control group. ##P < 0.01; ###P < 0.001 versus the LPS group. The Shapiro-Wilk test results showed that all the data are normally distributed (p > 0.05). [file 12974_2020_1836_MOESM1_ESM.tif]

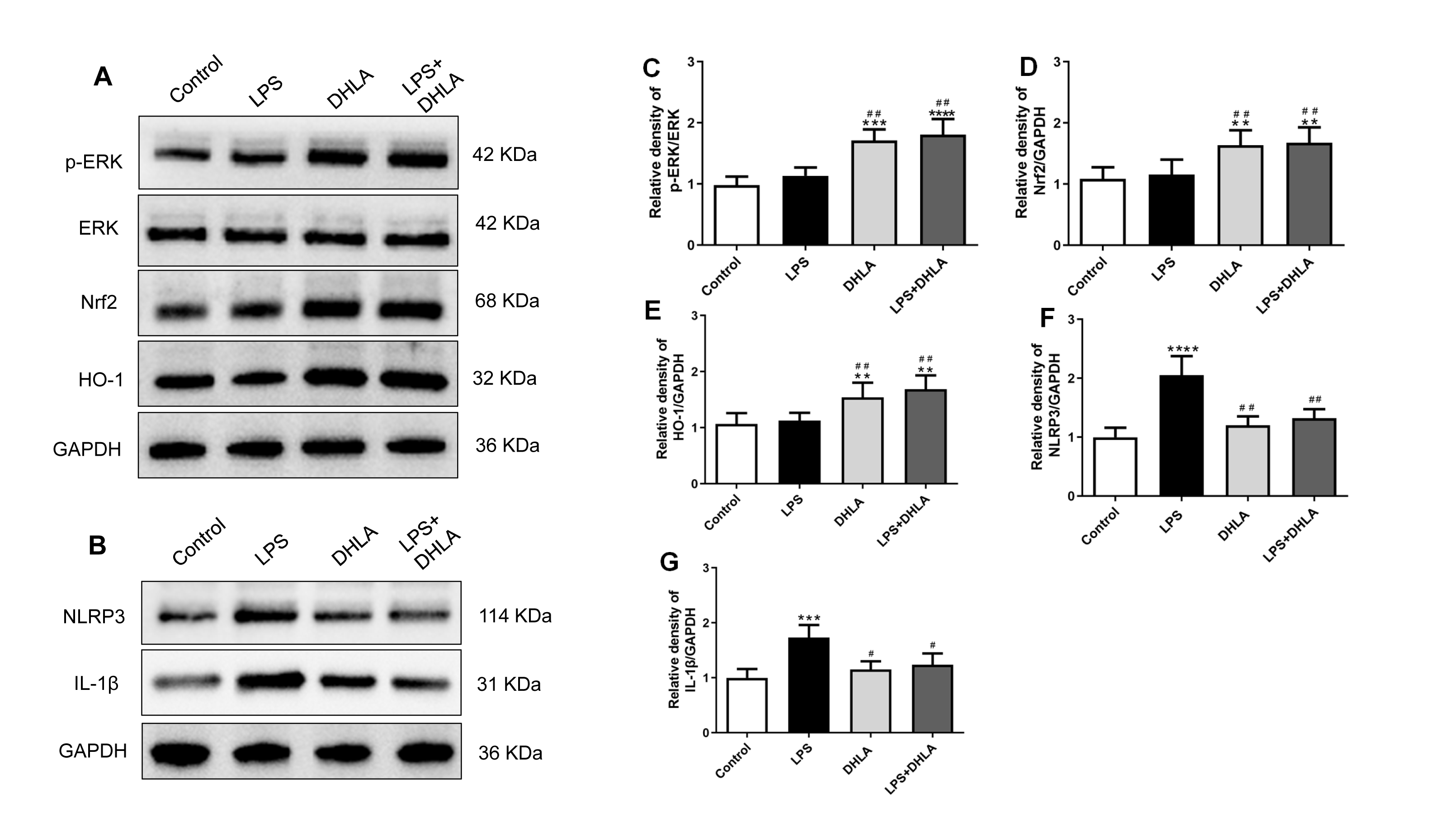

Supplement: Supplementary file 2 — Additional file 2: Figure S2. Effect of DHLA on ERK/Nrf2/HO-1/NLRP3/IL-1β signaling pathway in rats. a-b Representative Western blot bands in the hippocampal region. c-g Statistical graphs of relative protein expression of p-ERK/ERK (c), Nrf2 (d), HO-1 (e), NLRP3 (f), IL-1β (g). The data were expressed as means ± SEM (n=6). **P < 0.01; ***P < 0.001; ****P < 0.0001, versus the control group. #P < 0.05; ##P < 0.01, versus the LPS group. The Shapiro-Wilk test results showed that all the data are normally distributed (p > 0.05). [file 12974_2020_1836_MOESM2_ESM.tif]

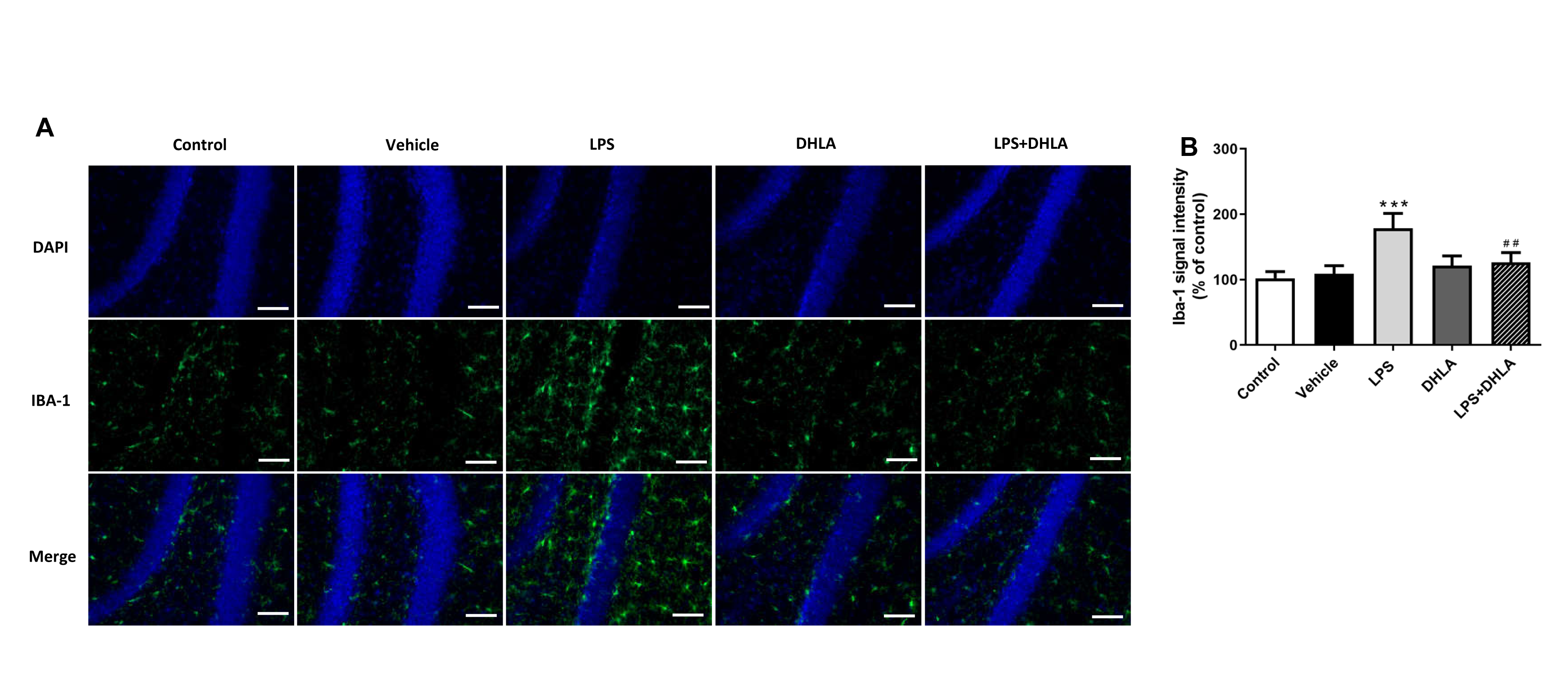

Supplement: Supplementary file 3 — Additional file 3: Figure S3. Effect of optimal dose of DHLA prevents LPS-induced increase of the microglial number. a Representative images of immunofluorescence assays of Iba1 in the hippocampus. Six micrographs from three rats per group were analyzed. b DHLA blocked the increased Iba-1 signal intensity induced by LPS. Scale bars represent 50 μm. The data were expressed as means ± SEM (n=6). ***P < 0.001, versus the control group. ##P < 0.01 versus the LPS group. The Shapiro-Wilk test results showed that all the data are normally distributed (p > 0.05). [file 12974_2020_1836_MOESM3_ESM.tif]
